# Serum ferritin critical threshold fluctuations predict antipsychotic treatment discontinuation in pediatric Tic disorders: a retrospective cohort study

**DOI:** 10.3389/fped.2026.1826108

**Published:** 2026-06-19

**Authors:** Linxia Li, Xianjin Yao, Jianing Dou, Xiaoping Qian, Zhanli Liu, Shasha Liu, Zelin Hao

**Affiliations:** 1 Department of Neurology, Hangzhou Children’s Hospital, Hangzhou, China; 2 Department of Traditional Chinese Medicine, Hangzhou Children’s Hospital, Hangzhou, China; 3 Department of Neurosurgery, The Affiliated Hospital of Hangzhou Normal University, Hangzhou, China

**Keywords:** antipsychotic agents, iron metabolism, serum ferritin, tic disorders, treatment discontinuation

## Abstract

**Objective:**

To determine whether dynamic fluctuations in serum ferritin below a critical threshold (30 ng/mL) independently predict antipsychotic treatment discontinuation in children with tic disorders, and to evaluate effect modification by antipsychotic class.

**Methods:**

A single-center retrospective cohort study analyzed electronic medical records (2022–2024) of 228 children (aged 6–16 years) with Diagnostic and Statistical Manual of Mental Disorders, Fifth Edition (DSM-5)-diagnosed tic disorders receiving first-line antipsychotics. Participants were stratified by ferritin fluctuation patterns: high-fluctuation (coefficient of variation [CV] ≥25% with ≥1 value <30 ng/mL; *n* = 78) vs. low-fluctuation (CV <25% or all values ≥30 ng/mL; *n* = 150). The primary outcome was treatment discontinuation. Cox regression models were sequentially adjusted for demographic/clinical factors and an extended set of covariates (including nutritional and socioeconomic proxies).

**Results:**

Over a 22.4-month median follow-up, high-fluctuation patients showed significantly higher discontinuation rates (60.3% vs. 24.0%; *P* < 0.001), with lack of efficacy being a more frequent reason in this group. After full adjustment, high ferritin fluctuation predicted a >2-fold discontinuation risk (hazard ratio [HR] = 2.24, 95% confidence interval [CI]: 1.43–3.52; *P* < 0.001), which remained significant after further adjustment for extended covariates (HR = 2.11, 95% CI: 1.34–3.33, *P* = 0.001). An exploratory analysis indicated that high fluctuation was also associated with a slower rate of tic symptom improvement over time (*P* = 0.002). Subgroup analysis revealed a stronger association with typical antipsychotics (HR = 4.12) vs. atypical agents (HR = 1.87; *P* interaction = 0.021). Results were robust across sensitivity analyses, including assessment of potential monitoring bias. Baseline ferritin <30 ng/mL alone was non-predictive (HR = 1.18; *P* = 0.42).

**Conclusion:**

Dynamic ferritin fluctuations below 30 ng/mL independently predict antipsychotic discontinuation and poorer symptomatic improvement in pediatric tic disorders, with heightened risk for typical antipsychotic users. Serial ferritin monitoring may identify high-risk patients for early intervention.

## 1 Introduction

Tic disorders are among the most common neurodevelopmental conditions in childhood, typically manifesting as sudden, recurrent, non-rhythmic motor or vocal tics. Epidemiological surveys estimate that up to 25% of children experience tics at some point during development, particularly between the ages of 5 and 12 years, though most cases are transient in nature (1). Chronic tic disorders, including Tourette syndrome (TS), are more persistent and affect approximately 0.6% of children worldwide (2). In the United States, recent Centers for Disease Control and Prevention (CDC) data show a TS prevalence of 1 in 162 children, with a marked male predominance (3). Importantly, comorbid neuropsychiatric conditions such as attention-deficit/hyperactivity disorder (ADHD), obsessive-compulsive disorder, and autism spectrum disorder occur in over 80% of patients with TS, contributing to significant functional impairment (4).

Iron is a vital cofactor in neurodevelopmental processes and plays an essential role in dopamine synthesis, a neurotransmitter critically involved in the pathophysiology of tic disorders. Serum ferritin, as a clinical surrogate for total body iron stores, is commonly used to assess iron status, with values below 30 ng/mL considered indicative of deficiency in the absence of inflammation (5). Several studies have identified lower ferritin levels in children with tic disorders compared to healthy controls, suggesting a potential relationship between iron metabolism and symptom severity (6). Specifically, iron acts as a crucial cofactor for tyrosine hydroxylase, the rate-limiting enzyme in dopamine synthesis, meaning that iron deficiency can directly impair dopaminergic neurotransmission within the basal ganglia circuits central to TS pathophysiology (7). This provides a strong biological basis for the clinical observations that link lower iron stores to more severe tic manifestations. For instance, children with TS and low ferritin levels were observed to have more severe tics, with symptom improvement following iron supplementation (8).

Despite these findings, prior research has largely relied on single-point ferritin measurements, failing to capture temporal variability and its clinical implications. Notably, serum ferritin is a dynamic biomarker that can fluctuate with physiological or pathological changes, and such variability may carry prognostic significance. This concept is supported by evidence from ADHD populations, where higher baseline ferritin levels were associated with increased risk of symptom exacerbation after stimulant withdrawal (9). Additionally, iron deficiency is known to modulate dopaminergic tone, which may influence treatment tolerability or response to antipsychotic medications used in tic management (10).

Given the potential clinical value of tracking iron status over time, we hypothesize that dynamic fluctuations in serum ferritin levels, particularly episodes falling below 30 ng/mL, may be associated with increased risk of treatment discontinuation in children with tic disorders. Therefore, the primary objective of this study is to investigate whether intra-individual ferritin variability predicts premature discontinuation of antipsychotic therapy in this population. Secondary aims include evaluating the influence of baseline ferritin, comorbidities, and medication type on the observed relationship. By exploring these associations, we aim to inform more personalized monitoring strategies and support early intervention in patients at risk of poor treatment adherence.

## 2 Methods

### 2.1 Study design

This single-center retrospective cohort study utilized electronic medical records (EMR) to investigate the association between dynamic fluctuations in serum ferritin levels at a critical threshold (<30 ng/mL) and the risk of treatment discontinuation in children diagnosed with tic disorders. Data were collected from January 1, 2022, to December 31, 2024, ensuring a minimum follow-up period of 24 months for all eligible participants. The study protocol was approved by the Ethics Committee of the Hangzhou Children's Hospital. A waiver of informed consent was granted in accordance with local regulations and the Declaration of Helsinki, as the research involved anonymized retrospective data analysis without direct patient intervention. All data were managed under strict confidentiality protocols to ensure patient privacy.

### 2.2 Study population

#### 2.2.1 Inclusion criteria

Patients were eligible if they met all of the following:

1. Age 6–16 years at treatment initiation;
2. Confirmed diagnosis of tic disorder per Diagnostic and Statistical Manual of Mental Disorders, 5th Edition (DSM-5) criteria for TS or persistent motor/vocal tic disorder (11);
3. Initiation of first-line pharmacotherapy (dopamine receptor antagonists: typical/atypical antipsychotics) between January 2022 and December 2024;
4. Availability of ≥1 baseline and ≥2 follow-up serum ferritin measurements (minimum interval: 3 months; maximum interval: 6 months);
5. Minimum documented follow-up duration of 6 months post-treatment initiation.

#### 2.2.2 Exclusion criteria

Patients were excluded for any of the following:

1. Comorbid neurodevelopmental disorders (autism spectrum disorder [ASD], intellectual disability) except ADHD or obsessive-compulsive disorder (OCD);
2. Iron supplementation within 3 months preceding baseline ferritin measurement or during follow-up;
3. Conditions affecting iron metabolism (chronic inflammatory diseases, hereditary hemochromatosis, or acute infections at sampling) (12);
4. Incomplete medical records (missing severity scores, treatment logs, or ≥50% of covariates);
5. Loss to follow-up before completing 6 months of therapy.

#### 2.2.3 Cohort assembly

The cohort was identified through systematic queries of the Hangzhou Children's Hospital EMR system using ICD-10 codes for tic disorders (F95.0–F95.2). Screening involved sequential filters: Initial eligibility: Age 6–16 years+tic disorder diagnosis+antipsychotic prescription; Ferritin data verification: Manual review of laboratory databases for ≥3 measurements; Covariate validation: Cross-checking of Yale Global Tic Severity Scale (YGTSS) scores (13), comorbidities, and treatment logs.

#### 2.2.4 Sample size justification

The sample size was estimated using the “powerSurvEpi” package in R 4.2. Based on a Cox proportional hazards model (*α* = 0.05, power = 80%), a target hazard ratio (HR) of 1.8 for treatment discontinuation in high-fluctuation group, and an anticipated event rate of 35% (5), a minimum of 214 participants was required. This accounted for 15% attrition due to data incompleteness.

### 2.3 Exposure definition

Serum ferritin fluctuations at the critical threshold (<30 ng/mL) served as the primary exposure. Measurements were standardized to fasting morning samples analyzed via chemiluminescent immunoassay (Architect i2000SR, Abbott Laboratories). Fluctuation magnitude was quantified using the coefficient of variation (CV = [standard deviation (SD)/Mean] × 100%). Participants were stratified into:

1. High-fluctuation group: CV ≥25% with ≥1 measurement <30 ng/mL (WHO-defined iron deficiency threshold in children (5));
2. Low-fluctuation group: CV <25% or all measurements ≥30 ng/mL.

#### 2.3.1 Exploratory stratification by mean serum ferritin level

To examine potential confounding across differing iron stores, we performed an exploratory *post-hoc* analysis stratifying participants by their overall iron status. This was based on the arithmetic mean of all serial ferritin measurements for each participant. The cohort was divided into three groups reflecting low, intermediate, and high iron stores:

1. Low mean ferritin group: Mean ferritin <30 ng/mL (*n* = 70). This threshold aligns with the WHO-defined cutoff for iron deficiency in children (5).
2. Intermediate mean ferritin group: 30 ng/mL ≤ Mean ferritin ≤50 ng/mL (*n* = 110). This range represents borderline to adequate iron stores. The upper limit of 50 ng/mL was chosen as it approximated the 75th percentile of the distribution in our cohort, ensuring clinically meaningful and comparably sized groups.
3. High mean ferritin group: Mean ferritin >50 ng/mL (*n* = 48). Participants in this group exhibited robust iron stores while remaining below the common threshold (100 ng/mL) indicative of probable concurrent inflammation.

This stratification was used solely for the descriptive comparison of baseline characteristics and to inform the consideration of covariates in modeling. All primary inferential analyses (e.g., Cox regression) remained based on the pre-specified exposure of ferritin fluctuation pattern (high vs. low), as defined in Section 2.3.

### 2.4 Outcome assessment and data collection

#### 2.4.1 Primary outcome

The primary outcome was treatment discontinuation, defined as meeting any of the following criteria: (1) Active cessation of first-line pharmacotherapy for >30 consecutive days without medical justification (e.g., symptom remission), verified through prescription renewal gaps in pharmacy dispensing records; (2) Permanent medication switch due to inadequate therapeutic response (≤25% YGTSS score reduction) or intolerable adverse events (AEs) documented in clinician progress notes; (3) Loss to follow-up with the last recorded status explicitly indicating treatment termination. Time-to-event was calculated from treatment initiation until discontinuation, with right-censoring applied to patients continuing therapy at study end (December 31, 2024).

#### 2.4.2 Covariate data collection

Baseline and longitudinal variables were systematically extracted from structured and unstructured EMR using REDCap electronic data capture tools. Demographic and clinical characteristics included age, sex, baseline YGTSS total score (abstracted from neurology assessments within ±7 days of treatment initiation), and comorbidities validated through ICD-10 codes, including attention-deficit/hyperactivity disorder (ADHD, F90.0), obsessive-compulsive disorder (OCD, F42), and additionally, anxiety disorders (F40-F42) and depressive disorders (F32-F33). The total number of these comorbid psychiatric conditions (0, 1, or ≥2) was calculated to indicate the overall comorbidity burden. To account for socioeconomic and nutritional factors, we also extracted the primary type of health insurance (categorized as urban basic medical insurance vs. others) and the presence of documented “picky eating,” “restrictive eating,” or “poor appetite” from clinical notes. Iron metabolism parameters encompassed baseline serum ferritin (closest measurement within 30 days pre-treatment), hemoglobin (Hb), and mean corpuscular volume (MCV), with all hematological assays performed using standardized methods (Abbott ARCHITECT ci16200 analyzers; coefficient of variation <5%). Treatment-related variables covered antipsychotic class (typical: haloperidol, pimozide; atypical: risperidone, aripiprazole), starting dose (mg/kg/day), and dose adjustment frequency. Healthcare engagement during follow-up was assessed by the total number of outpatient visits to neurology or psychiatry. Ferritin measurements required confirmation of fasting status and absence of acute inflammation (C-reactive protein [CRP] <5 mg/L).

#### 2.4.3 Safety monitoring

Treatment-emergent adverse events (TEAEs) were identified through multimodal surveillance: (1) Active AE documentation in neurology clinic notes using Medical Dictionary for Regulatory Activities (MedDRA v26.0) preferred terms; (2) Systematic laboratory alert flags for metabolic abnormalities (prolactin >25 ng/mL, fasting glucose >126 mg/dL, or QTc prolongation >450 ms); (3) Unplanned hospitalizations linked to antipsychotic use via ICD-10 codes (e.g., T43.3X5A for neuroleptic poisoning); (4) Discontinuation records explicitly citing AEs as the reason. Severity was graded per Common Terminology Criteria for Adverse Events (CTCAE v6.0) (14).

#### 2.4.4 Data quality assurance

To mitigate retrospective data limitations, a three-tier validation protocol was implemented: (1) Automated logic checks for temporal consistency (e.g., ferritin measurements spaced 3–6 months apart); (2) Independent dual abstraction for 15% randomly selected records (*κ* > 0.85 for key variables); (3) Endpoint adjudication committee review of ambiguous discontinuation events. Ferritin assay quality control followed CLIA guidelines with biannual calibration verification. Missing covariate data (<5% overall) were addressed via multiple imputation using chained equations (MICE).

### 2.5 Statistical analysis

Statistical analyses were performed using SPSS 26.0 (IBM Corp.) and R 4.2 (with the survival, cmprsk, and lme4 packages). Continuous variables were expressed as mean ± SD or median [interquartile range (IQR)] based on normality (Shapiro–Wilk test), with between-group comparisons using independent *t*-tests, Mann–Whitney *U*-tests, one-way ANOVA, or Kruskal–Wallis tests as appropriate. Categorical variables were reported as frequencies (%) and analyzed via *χ*^2^ or Fisher's exact tests. Trends across ordered groups were assessed using the linear-by-linear association *χ*^2^ test.

The primary time-to-event analysis employed Kaplan–Meier curves with log-rank tests to compare treatment discontinuation rates between ferritin fluctuation groups. Cox proportional hazards models generated hazard ratios (HRs) and 95% confidence intervals (CIs) in sequential adjustments: Model 1 (unadjusted), Model 2 (adjusted for age, sex, baseline YGTSS), Model 3 (additionally adjusted for antipsychotic class, ADHD/OCD comorbidity, and baseline ferritin), and an extended Model 4 (further adjusted for documented picky eating, urban basic medical insurance, total outpatient visits, and total psychiatric comorbidity burden). Proportional hazards assumptions were verified using Schoenfeld residuals.

To address specific reviewer queries, several additional analyses were conducted. First, the reasons for treatment discontinuation were analyzed by fitting Fine-Gray subdistribution hazard models to account for the competing risks of discontinuation due to lack of efficacy vs. adverse events. Second, an exploratory linear mixed-effects model was used to analyze the longitudinal trajectory of YGTSS total scores, with fixed effects for time, ferritin fluctuation group, their interaction, and covariates (age, sex, antipsychotic class, baseline YGTSS), and random participant intercepts. Third, *post-hoc* descriptive comparisons were performed by stratifying the cohort based on mean serum ferritin level (low: <30 ng/mL; intermediate: 30–50 ng/mL; high: >50 ng/mL) and by intensity of monitoring (higher: ≥5 measurements; lower: ≤4 measurements) to assess group differences in baseline characteristics and exposure distribution.

Sensitivity analyses for the primary outcome included: (1) redefining high fluctuation using absolute standard deviation (SD ≥8 ng/mL with ≥1 value <30 ng/mL) instead of CV; (2) excluding patients with baseline ferritin >100 ng/mL; (3) competing risks analysis for loss to follow-up; and (4) repeating the primary Cox regression in the subgroup of patients with lower monitoring intensity (≤4 ferritin measurements).

Statistical significance was set at *α* = 0.05 (two-tailed). For the primary analysis and pre-specified sensitivity analyses, no multiplicity adjustment was applied. Bonferroni correction was considered for multiple comparisons within exploratory and *post-hoc* analyses.

## 3 Results

### 3.1 Study population and baseline characteristics

**Figure 1:**
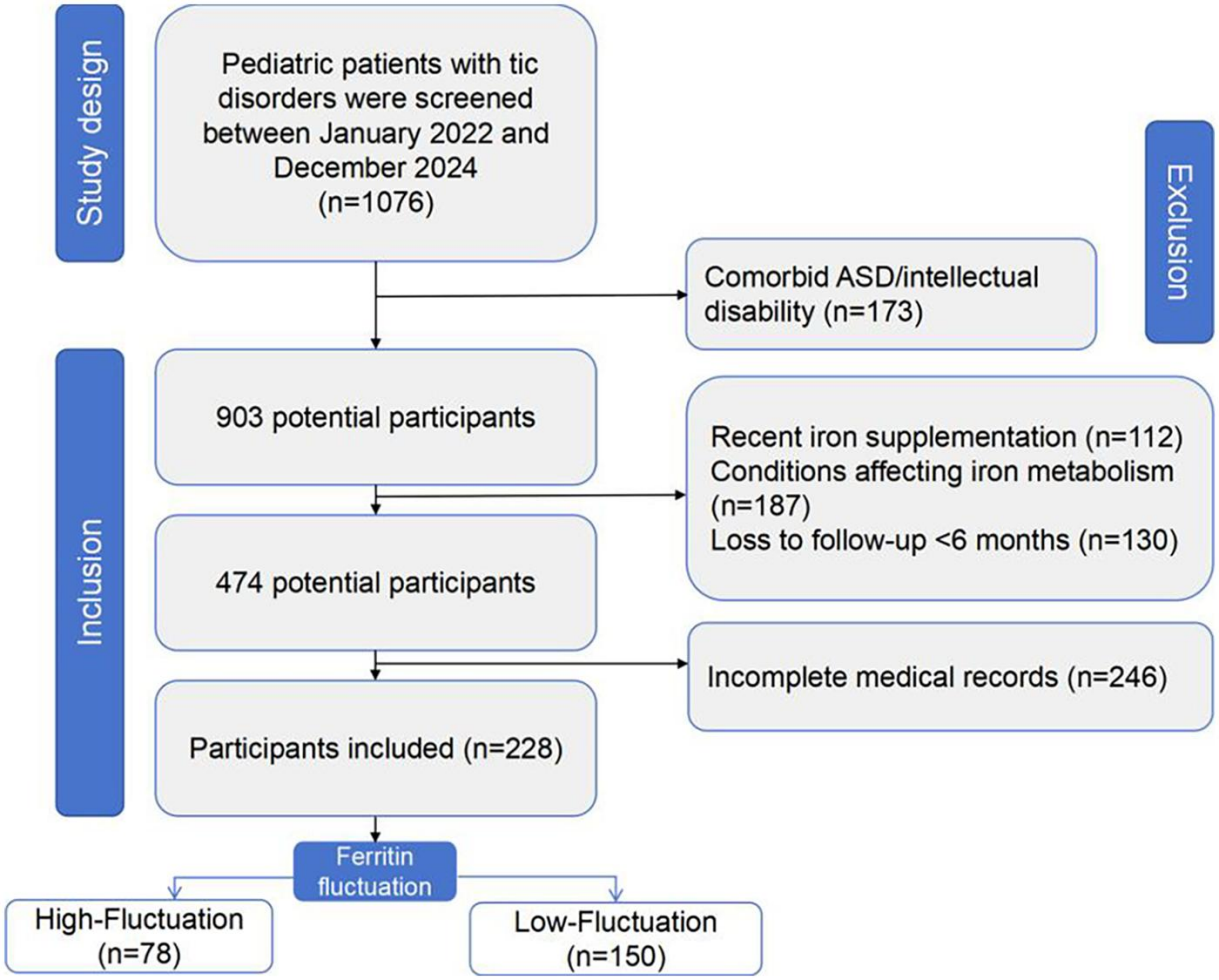
Inclusion and exclusion flowchart.

A total of 1,243 pediatric patients with tic disorders were screened between January 2022 and December 2024. After applying inclusion/exclusion criteria, 228 participants were enrolled (Figure 1). The cohort comprised 165 males (72.4%) and 63 females (27.6%), with a mean age of 10.3 ± 2.7 years. Stratification by ferritin fluctuation patterns identified 78 patients (34.2%) in the high-fluctuation group (CV ≥25% with ≥1 value <30 ng/mL) and 150 (65.8%) in the low-fluctuation group (Figure 1). The high-fluctuation group demonstrated significantly lower baseline ferritin levels (25.6 ± 8.3 ng/mL vs. 41.2 ± 13.7 ng/mL; *P* < 0.001), higher baseline YGTSS scores (64.8 ± 11.2 vs. 52.3 ± 10.6; *P* < 0.001), and increased prevalence of severe tics (YGTSS >60: 65.4% vs. 28.7%; *P* < 0.001). No significant differences were observed in antipsychotic class distribution or starting dosages between groups. The groups were also comparable in terms of age, sex, tic subtype, and the prevalence of ADHD, OCD, and positive family history. Regarding the additionally extracted covariates, the high-fluctuation group had a nominally lower proportion of participants with urban basic medical insurance and a significantly higher prevalence of documented picky eating (*P* = 0.012). The overall psychiatric comorbidity burden and the intensity of healthcare engagement, as measured by outpatient visit frequency, did not differ significantly between the two groups (Table 1).

**Table 1:** Baseline characteristics of study population stratified by ferritin fluctuation patterns.

Characteristic	Overall (*n* = 228)	High-Fluctuation (*n* = 78)	Low-Fluctuation (*n* = 150)	Statistic	*P*-value
Demographics					
Age, years	10.3 ± 2.7	10.1 ± 2.9	10.4 ± 2.5	*t* = 0.83	0.407
Male sex	165 (72.4%)	57 (73.1%)	108 (72.0%)	*Χ*^2^ = 0.03	0.861
Clinical Features					
Tic subtype:				FET	0.213
Tourette syndrome	124 (54.4%)	47 (60.3%)	77 (51.3%)		
Chronic motor tic	82 (36.0%)	24 (30.8%)	58 (38.7%)		
Chronic vocal tic	22 (9.6%)	7 (9.0%)	15 (10.0%)		
Baseline YGTSS total	56.4 ± 12.1	64.8 ± 11.2	52.3 ± 10.6	*t* = 8.47	<0.001
Severe tics (YGTSS >60)	95 (41.7%)	51 (65.4%)	43 (28.7%)	χ^2^ = 28.5	<0.001
Comorbidities:					
ADHD	142 (62.3%)	53 (67.9%)	89 (59.3%)	Χ^2^ = 1.71	0.191
OCD	87 (38.2%)	33 (42.3%)	54 (36.0%)	χ^2^ = 0.89	0.346
Anxiety disorder	46 (20.2%)	19 (24.4%)	27 (18.0%)	χ^2^ = 1.31	0.252
Depressive disorder	22 (9.6%)	9 (11.5%)	13 (8.7%)	χ^2^ = 0.50	0.478
Total psychiatric comorbidities (0/1/≥2)	89/98/41 (39.0%/43.0%/18.0%)	25/36/17 (32.1%/46.2%/21.8%)	64/62/24 (42.7%/41.3%/16.0%)	χ^2^ = 2.34	0.310
Positive family history	74 (32.5%)	29 (37.2%)	45 (30.0%)	χ^2^ = 1.23	0.268
Additional Covariates					
Urban basic medical insurance	158 (69.3%)	49 (62.8%)	109 (72.7%)	χ^2^ = 2.38	0.123
Documented picky eating	68 (29.8%)	32 (41.0%)	36 (24.0%)	χ^2^ = 6.30	0.012
Outpatient visits, n	8 [5–12]	9 [6–13]	8 [5–11]	*U* = 5,220	0.102
Iron Metabolism					
Baseline ferritin, ng/mL	35.8 ± 14.2	25.6 ± 8.3	41.2 ± 13.7	*t* = 9.12	<0.001
Hemoglobin, g/dL	13.1 ± 1.4	12.7 ± 1.3	13.3 ± 1.4	*t* = 3.28	0.001
MCV, fL	85.2 ± 4.8	83.4 ± 4.5	86.1 ± 4.6	*t* = 4.13	<0.001
Treatment Parameters					
Antipsychotic class:				χ^2^ = 0.42	0.517
Atypical (risperidone/aripiprazole)	193 (84.6%)	64 (82.1%)	129 (86.0%)		
Typical (haloperidol/pimozide)	35 (15.4%)	14 (17.9%)	21 (14.0%)		
Starting dose, mg/kg/day	0.48 [0.3–0.7]	0.52 [0.35–0.75]	0.46 [0.3–0.65]	*U* = 5,241	0.089

Values presented as mean ± SD, n (%), or median [IQR].

YGTSS, Yale Global Tic Severity Scale; ADHD, attention-deficit/hyperactivity disorder; OCD, obsessive-compulsive disorder; MCV, mean corpuscular volume; FET, Fisher's exact test. **Statistical tests:** Independent *t*-test (normal variables), Mann–Whitney U (non-normal), χ^2^ or FET (categorical).

### 3.2 Ferritin fluctuation profiles

Dynamic ferritin monitoring revealed distinct fluctuation patterns between groups (Table 2). Participants underwent a median of 4 [IQR: 3–5] serum ferritin measurements over 18.2 ± 5.3 months. The high-fluctuation group (*n* = 78) demonstrated significantly greater variability (CV: 32.4% ± 6.8% vs. 14.7% ± 5.2%; *P* < 0.001), with 92.3% exhibiting ≥2 measurements <30 ng/mL. Conversely, 86.0% of the low-fluctuation group maintained all values ≥30 ng/mL. Clinically implausible values (<5 ng/mL or >500 ng/mL) were absent, confirming data integrity.

**Table 2:** Serum ferritin fluctuation characteristics.

Parameter	Overall (*n* = 228)	High-Fluctuation (*n* = 78)	Low-Fluctuation (*n* = 150)	Statistic	*P*-value
Monitoring Parameters					
Follow-up duration, months	18.2 ± 5.3	17.9 ± 5.1	18.4 ± 5.4	*t* = 0.68	0.495
Number of ferritin measurements	4 [3–5]	4 [4–5]	4 [3–5]	*U* = 5,421	0.114
Measurement interval, months	4.3 ± 1.1	4.2 ± 1.0	4.4 ± 1.2	*t* = 1.22	0.225
Ferritin Dynamics					
Mean ferritin, ng/mL	33.7 ± 11.9	27.1 ± 7.8	37.2 ± 11.6	*t* = 7.63	<0.001
Ferritin SD, ng/mL	8.4 ± 4.3	12.1 ± 3.9	6.5 ± 2.7	*t* = 13.2	<0.001
Coefficient of variation (CV), %	24.9 ± 11.7	32.4 ± 6.8	14.7 ± 5.2	*t* = 21.4	<0.001
Minimum ferritin, ng/mL	26.5 [18–34]	19 [14–25]	31 [26–35]	*U* = 1,285	<0.001
Maximum ferritin, ng/mL	45 [36–58]	39 [31–47]	48 [40–62]	*U* = 3,014	<0.001
Threshold Violations					
Patients with ≥1 value <30 ng/mL	123 (54.0%)	78 (100%)	45 (30.0%)	FET	<0.001
Measurements <30 ng/mL, % of total	29.1%	52.4%	12.7%	χ^2^ = 318	<0.001

Values presented as mean ± SD, median [IQR], or *n* (%). SD, standard deviation; CV, coefficient of variation; FET, Fisher's exact test. Statistical tests: Independent *t*-test (normal variables), Mann–Whitney U (non-normal), χ^2^ or FET (categorical).

### 3.3 Treatment discontinuation risk analysis

**Figure 2:**
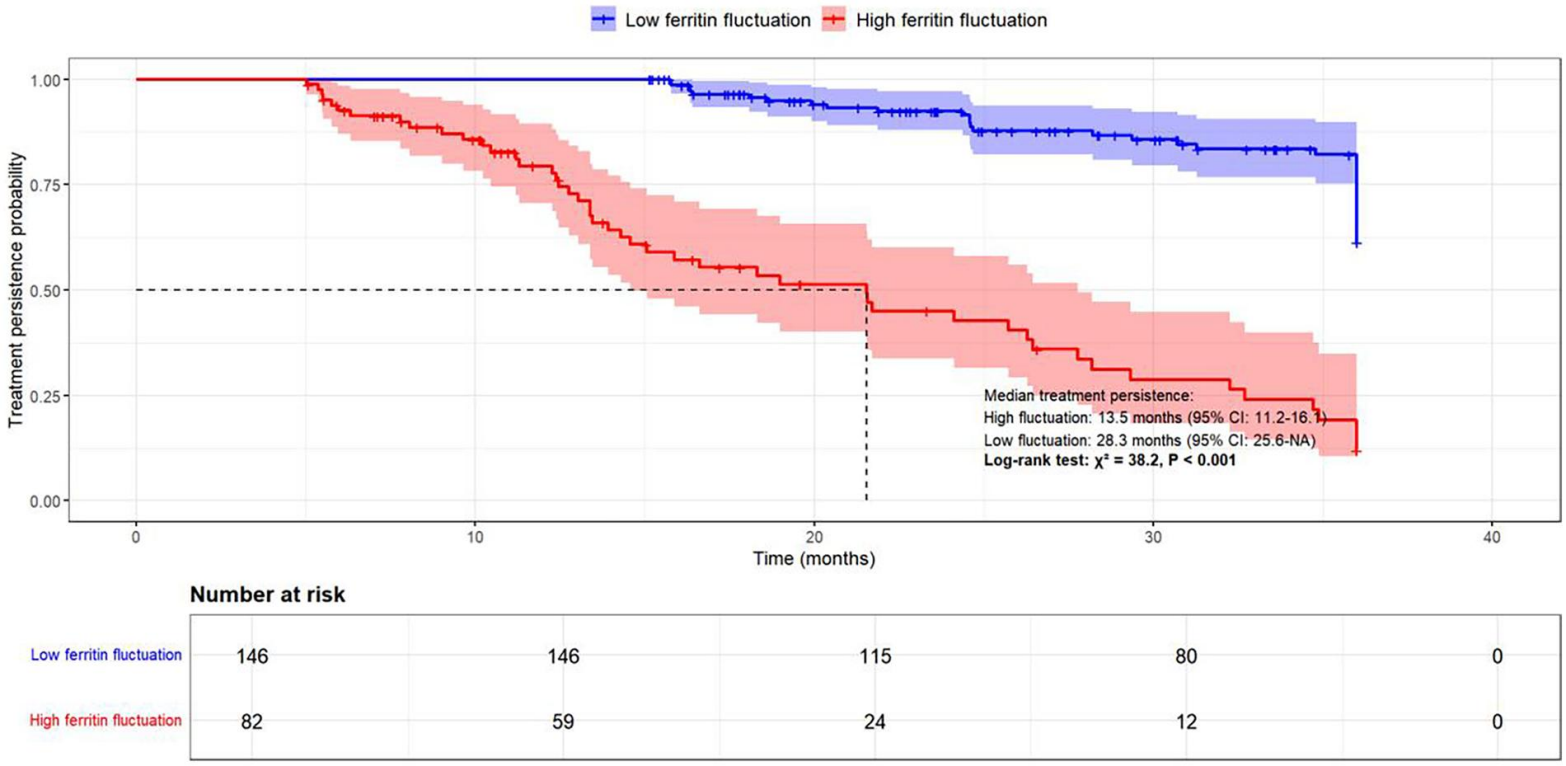
Kaplan–meier analysis of treatment persistence by ferritin fluctuation group. The graph shows significantly shorter treatment persistence in patients with high ferritin fluctuation (red line) compared to those with low fluctuation (blue line). Median treatment persistence was 13.5 months (95% CI: 11.2–16.1) in the high-fluctuation group vs. 28.3 months (95% CI: 25.6-NA) in the low-fluctuation group. Shaded areas represent 95% confidence intervals. The difference between groups was statistically significant (log-rank *χ*^2^ = 38.2, *P* < 0.001). The risk table shows the number of patients still on treatment at each time point. NA = not attained.

Over a median follow-up of 22.4 months (IQR: 18.6–26.1), 83 treatment discontinuation events occurred (36.4% overall incidence). Among these, 45 (54.2%) were attributed to lack of efficacy (≤25% reduction in YGTSS score) and 38 (45.8%) to intolerable adverse events. The high-ferritin-fluctuation group demonstrated significantly higher discontinuation rates (60.3% vs. 24.0%; *P* < 0.001). Notably, the distribution of reasons differed between groups: lack of efficacy accounted for a higher proportion of discontinuations in the high-fluctuation group (66.0%, 31/47) compared to the low-fluctuation group (38.9%, 14/36), whereas adverse events were more frequently the reason in the low-fluctuation group (61.1%, 22/36 vs. 34.0%, 16/47; *P* = 0.015 for difference in reason distribution). Kaplan–Meier analysis revealed accelerated treatment failure in this group (log-rank *χ*² = 38.2, *P* < 0.001), with median treatment persistence of 13.5 months (95% CI: 11.2–16.1) vs. 28.3 months (95% CI: 25.6-NA) in the low-fluctuation group (Figure 2). Cox regression confirmed ferritin fluctuations as an independent predictor: after adjustment for age, sex, and baseline tic severity (Model 2), and further adjustment for antipsychotic class, ADHD/OCD comorbidity, and baseline ferritin (Model 3), high-fluctuation patients faced a greater than two-fold discontinuation risk (Model 3 HR = 2.24, 95% CI: 1.43–3.52, *P* < 0.001). To comprehensively address potential confounding from variables that differed at baseline or are clinically relevant, we fitted an extended model (Model 4) that additionally included documented picky eating, urban basic medical insurance status, total number of outpatient visits, and total psychiatric comorbidity burden. The association between high ferritin fluctuation and treatment discontinuation remained significant and materially unchanged (HR = 2.11, 95% CI: 1.34–3.33, *P* = 0.001). Baseline ferritin <30 ng/mL alone was not predictive in any adjusted model (Model 4 HR = 1.15, *P* = 0.47) (Table 3).

**Table 3:** Cox proportional hazards analysis for treatment discontinuation.

Variable	Model 1: Unadjusted HR (95% CI)	*P*-value	Model 2: Partially Adjusted^a^ HR (95% CI)	*P*-value	Model 3: Fully Adjusted^b^ HR (95% CI)	*P*-value	Model 4: Extensively Adjusted^c^ HR (95% CI)	*P*-value
Primary Exposure								
High ferritin fluctuation	2.89 (1.87–4.48)	<0.001	2.65 (1.70–4.13)	<0.001	2.24 (1.43–3.52)	<0.001	2.11 (1.34–3.33)	0.001
Covariates								
Age (per 1-year increase)	–	–	0.93 (0.85–1.02)	0.116	0.94 (0.86–1.03)	0.174	0.93 (0.85–1.02)	0.148
Male sex	–	–	1.27 (0.76–2.12)	0.362	1.31 (0.78–2.20)	0.312	1.29 (0.76–2.19)	0.341
Baseline YGTSS (per 5-point)	–	–	1.19 (1.07–1.32)	0.001	1.14 (1.03–1.27)	0.015	1.13 (1.01–1.26)	0.027
ADHD comorbidity	–	–	–	–	1.38 (0.85–2.23)	0.192	1.34 (0.82–2.18)	0.242
OCD comorbidity	–	–	–	–	0.87 (0.54–1.40)	0.567	0.85 (0.53–1.37)	0.512
Typical antipsychotic use	–	–	–	–	1.52 (0.88–2.63)	0.132	1.48 (0.85–2.57)	0.165
Baseline ferritin (per 10 ng/mL)	–	–	–	–	0.95 (0.79–1.14)	0.568	0.96 (0.80–1.15)	0.633
Documented picky eating	–	–	–	–	–	–	1.22 (0.77–1.92)	0.398
Urban basic medical insurance	–	–	–	–	–	–	0.89 (0.56–1.42)	0.632
Total outpatient visits (per 5 visits)	–	–	–	–	–	–	1.05 (0.90–1.22)	0.541
Total psychiatric comorbidities (≥2 vs. 0/1)	–	–	–	–	–	–	1.18 (0.73–1.90)	0.502

a Model 2 adjusted for age, sex, baseline YGTSS.

b Model 3 additionally adjusted for antipsychotic class, ADHD/OCD comorbidity, and baseline ferritin.

c Model 4 adjusted for all variables in Model 3 plus documented picky eating, urban basic medical insurance, total outpatient visits, and total psychiatric comorbidity burden.

HR, hazard ratio; CI, confidence interval; YGTSS, Yale Global Tic Severity Scale. Proportional hazards assumption met (global Schoenfeld residual *P* = 0.58).

### 3.4 Subgroup analyses

**Figure 3:**
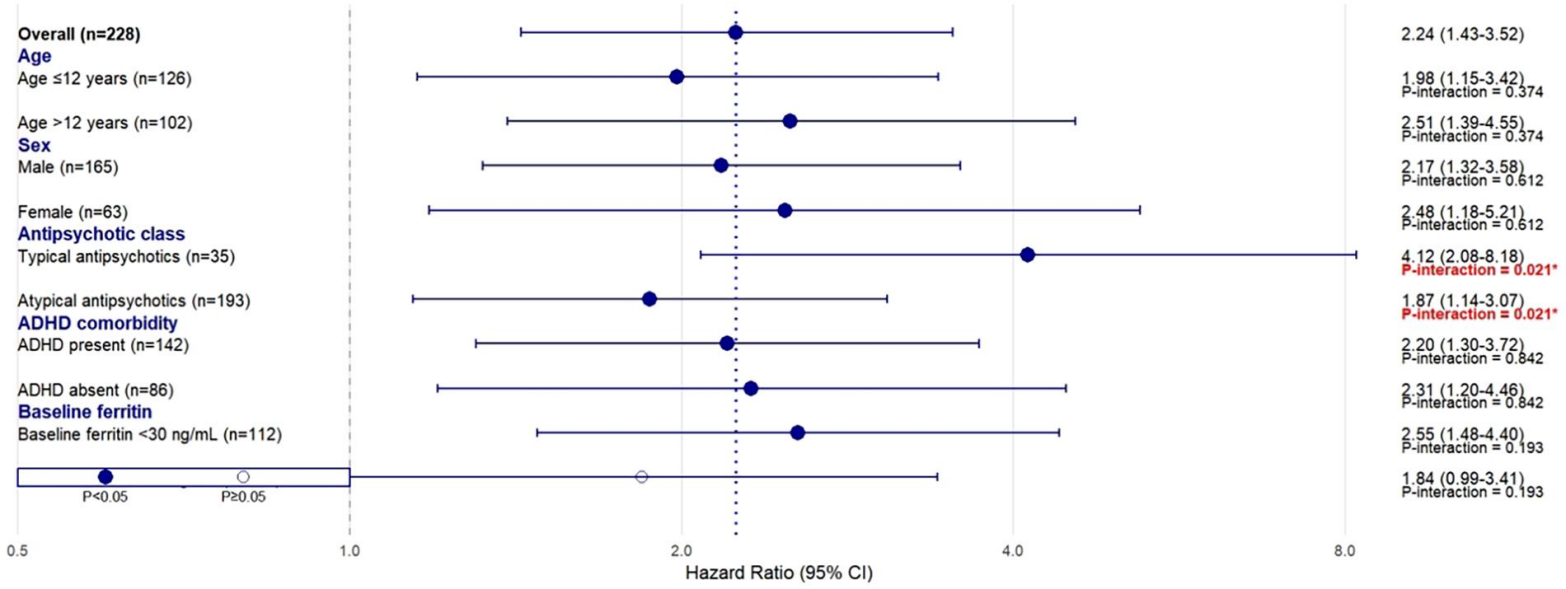
Subgroup analysis of ferritin fluctuation effect on antipsychotic treatment discontinuation. Hazard ratios with 95% confidence intervals are shown for different patient subgroups. The dotted vertical line represents the overall hazard ratio (2.24). Solid circles indicate statistically significant associations (*P* < 0.05); open circles indicate non-significant associations (*P* ≥ 0.05). Asterisk (*) denotes statistically significant interaction (*P* < 0.05). All models were adjusted for age, sex, baseline YGTSS score, antipsychotic class (when not the subgroup variable), ADHD/OCD comorbidity, and baseline ferritin level.

Subgroup analyses revealed significant effect modification by antipsychotic class (Pinteraction=0.021), with the ferritin fluctuation-discontinuation association being markedly stronger in patients receiving typical antipsychotics (HR = 4.12, 95% CI: 2.08–8.18) compared to atypical agents (HR = 1.87, 95% CI: 1.14–3.07). The association remained consistent across age and sex subgroups (all Pinteraction>0.05), though adolescents (>12 years) showed nominally higher risk (HR = 2.51 vs. 1.98 in ≤12 years) (Figure 3). Notably, the elevated risk persisted regardless of ADHD comorbidity status, refuting confounding by this common co-occurrence.

### 3.5 Sensitivity analyses

Sensitivity analyses confirmed the robustness of the primary findings (Table 4). When redefining high fluctuation using absolute standard deviation (SD ≥8 ng/mL with ≥1 value <30 ng/mL), the discontinuation risk remained elevated (HR = 2.07, 95% CI: 1.31–3.27). Exclusion of patients with potential inflammatory confounders (baseline ferritin >100 ng/mL, *n* = 15) strengthened the association (HR = 2.41, 95% CI: 1.52–3.83). Competing risks analysis accounting for loss to follow-up yielded nearly identical results (subdistribution HR = 2.19, 95% CI: 1.40–3.43), confirming minimal bias from administrative censoring.

**Table 4:** Sensitivity analyses of ferritin fluctuation effect on treatment discontinuation.

Analysis Approach	High-Fluctuation Group	Events in High-Fluctuation Group	Adjusted HR^a^ (95% CI)	*P*-value
Primary Analysis (CV-based)	78	47 (60.3%)	2.24 (1.43–3.52)	<0.001
Alternative Definition				
SD ≥8 ng/mL +<30 ng/mL	81	47 (58.0%)	2.07 (1.31–3.27)	0.002
Confounder Exclusion				
Baseline ferritin >100 ng/mL excluded	76	47 (61.8%)	2.41 (1.52–3.83)	<0.001
Competing Risks Model^b^	78	47 (60.3%)	sHR = 2.19 (1.40–3.43)	<0.001
Analysis in lower-monitoring-intensity subgroup (≤4 measurements)	38	22 (57.9%)	2.05 (1.18–3.58)	0.011

a Cox models adjusted for age, sex, baseline YGTSS, antipsychotic class, ADHD/OCD comorbidity, and baseline ferritin.

b Fine-Gray subdistribution hazard model accounting for loss to follow-up as competing risk.

HR, hazard ratio; sHR, subdistribution hazard ratio; CI, confidence interval.

In an exploratory analysis were stratified into three groups based on their mean serum ferritin level across all measurements: low mean ferritin (<30 ng/mL, *n* = 70), intermediate mean ferritin (30–50 ng/mL, *n* = 110), and high mean ferritin (>50 ng/mL, *n* = 48). Baseline characteristics across these groups are presented in Supplementary Table S1. Consistent with the primary fluctuation-based analysis, the low mean ferritin group exhibited the highest baseline tic severity (YGTSS total: 62.8 ± 11.8), the highest prevalence of severe tics (YGTSS >60: 64.3%), and the highest rate of documented picky eating (42.9%), with a statistically significant trend across groups (all *P* for trend <0.01). The groups did not differ significantly in age, sex distribution, antipsychotic class, or starting dose. This *post-hoc* stratification confirms that lower iron stores are associated with a more severe clinical profile and identifies specific covariates that were subsequently included in the extended adjustment model (Model 4, Table 3).

Additionally, to assess potential selection bias arising from differential frequency of ferritin monitoring, we compared baseline characteristics and the distribution of high ferritin fluctuation between participants with higher (≥5 measurements, *n* = 112) vs. lower (≤4 measurements, *n* = 116) monitoring intensity. The two groups were balanced on most key clinical variables including baseline tic severity (all *P* > 0.05, Supplementary Table S2). The proportion of patients with high ferritin fluctuation did not differ between monitoring intensity groups (35.7% vs. 32.8%, *P* = 0.654). Moreover, when the primary Cox model was restricted to the lower-intensity monitoring subgroup, the association remained significant (HR = 2.05, 95% CI: 1.18–3.58), consistent with the main analysis.

### 3.6 Exploratory analysis: association between ferritin fluctuation and tic severity trajectory

To investigate whether dynamic ferritin fluctuations influence the clinical course of tic symptoms, we performed an exploratory longitudinal analysis of YGTSS scores over time using a linear mixed-effects model. This model adjusted for baseline YGTSS, age, sex, antipsychotic class, and follow-up time, with random intercepts for participants to account for repeated measures. The primary term of interest was the interaction between ferritin fluctuation group (high vs. low) and time since treatment initiation (months).

Over the median follow-up of 22.4 months, participants contributed a total of 892 YGTSS assessments (mean 3.9 per participant). The model revealed a statistically significant interaction between ferritin fluctuation group and time (*β* = 1.82, 95% CI: 0.67–2.97, *P* = 0.002). This indicates that the rate of improvement in tic severity differed significantly between groups. Specifically, the high-fluctuation group exhibited a slower rate of symptom reduction compared to the low-fluctuation group. Estimated marginal means derived from the model illustrate this divergence (Supplementary Table S3). For example, at 18 months, the adjusted mean YGTSS was 46.3 (95% CI: 43.6–49.0) in the high-fluctuation group vs. 36.1 (95% CI: 34.0–38.2) in the low-fluctuation group. Full model parameters are presented in Supplementary Table S4. These findings suggest that dynamic instability in serum ferritin is associated not only with a higher risk of treatment discontinuation but also with a less favorable symptomatic trajectory among children who remain on therapy.

## 4 Discussion

The present study demonstrated that dynamic fluctuations in serum ferritin levels, particularly when levels fall below the critical threshold of 30 ng/mL, are independently associated with an increased risk of treatment discontinuation in children with tic disorders receiving antipsychotic therapy. This association remained significant even after adjusting for demographic and clinical covariates, including baseline tic severity and comorbidities. Crucially, this association proved robust to extensive adjustment for additional potential confounders such as nutritional risk (picky eating) and socioeconomic proxies, and was not explained by differential intensity of clinical monitoring. These findings highlight the potential value of longitudinal monitoring of ferritin variability as a predictive biomarker for treatment adherence risk in this population.

Our data revealed that patients exhibiting higher intra-individual variability in ferritin (CV ≥25%) were more likely to discontinue treatment prematurely compared to those with stable ferritin levels, despite comparable antipsychotic exposure. A finer analysis of discontinuation reasons provided further insight: the high-fluctuation group was disproportionately more likely to stop treatment due to perceived lack of efficacy rather than adverse events. This pattern aligns with previous findings indicating that iron metabolism disturbances may influence neuropsychiatric symptomatology and treatment response. For instance, a large cross-sectional study from China involving over 1,600 children with tic disorders reported lower mean ferritin levels in patients compared to healthy controls, although the study did not assess dynamic changes or their clinical implications (15). Our exploratory analysis based on mean ferritin levels corroborates this, showing a clear gradient where lower average iron stores are associated with a more severe clinical profile at baseline.

Moreover, our results are consistent with the work of Ghosh and colleagues, who observed that children with Tourette syndrome and lower ferritin levels tended to experience more severe tics and showed symptomatic improvement after iron supplementation (8). However, our study extends these findings by focusing not merely on absolute ferritin levels but on variability over time, suggesting that fluctuations below a critical threshold may be more clinically relevant than a single measurement. This is further supported by our exploratory longitudinal analysis, which found that high ferritin fluctuation was associated with a significantly slower rate of tic symptom improvement over time, independent of treatment discontinuation. This indicates that iron instability may negatively impact the core clinical trajectory, offering a plausible explanation for the higher rate of efficacy-related discontinuation.

Mechanistically, iron plays a critical role in dopamine metabolism and neurotransmitter regulation, both of which are implicated in tic disorders. Dysregulated iron homeostasis may lead to impaired dopamine signaling, thereby exacerbating tic severity and possibly contributing to treatment intolerance or non-adherence. This is supported by earlier neuroimaging studies linking low iron stores with altered basal ganglia volume and function in tic disorder patients (16). Additionally, animal models have shown that iron deficiency can impair dopaminergic neurotransmission (17, 18), potentially explaining heightened motor activity and decreased pharmacological tolerance. The stronger association we observed between ferritin fluctuation and discontinuation in patients prescribed typical antipsychotics may stem from this compromised dopaminergic system being less able to tolerate the potent receptor blockade of typical agents, potentially exacerbating both limited efficacy and side effects.

The findings of our study are strongly supported by a body of evidence linking iron homeostasis to the stability of the dopaminergic system, which is the primary target of antipsychotic medications. Mechanistically, iron deficiency has been demonstrated to decrease the density of dopamine D1 and D2 receptors in critical brain regions, which may blunt the therapeutic efficacy of these agents by reducing their intended binding sites (19). Furthermore, the interplay between low iron status and antipsychotic exposure can create a state of heightened neurobiological vulnerability. Preclinical models have shown that iron deficiency significantly exacerbates the supersensitivity of dopamine D2 receptors induced by neuroleptics (20). This heightened supersensitivity is a key mechanism implicated in the development of motor side effects, such as akathisia, which are common reasons for treatment discontinuation. This aligns with clinical meta-analytic data confirming that patients experiencing akathisia have significantly lower serum iron levels (21). Moreover, in pediatric cohorts receiving risperidone, lower ferritin levels were correlated with greater weight gain and poorer control of disruptive behaviors, suggesting a diminished overall treatment response (22). Collectively, these findings provide a robust biological rationale for our results, indicating that poor iron status compromises treatment by both limiting drug efficacy and increasing susceptibility to intolerable side effects.

The elevated treatment discontinuation risk observed in the high-fluctuation group may also reflect broader physiological instability. In line with our findings, Rosenau et al. reported that children with ADHD who had higher baseline ferritin levels were more vulnerable to symptom exacerbation following methylphenidate withdrawal, underscoring ferritin's role in modulating treatment response and neurobehavioral stability (9).

From a clinical perspective, the predictive utility of ferritin variability is particularly valuable given the non-invasive, cost-effective nature of ferritin testing. Routine monitoring of ferritin trends may enable early identification of patients at risk of poor adherence, guiding proactive iron supplementation or more frequent follow-up. Evidence from trials in other pediatric populations, such as those with autism spectrum disorder and restless leg syndrome, shows that intravenous iron supplementation (e.g., ferric carboxymaltose) can significantly improve symptoms in children with ferritin <30 ng/mL, suggesting a potential interventional strategy for tic disorder patients as well (23).

Our subgroup analysis also suggested that the association between ferritin fluctuations and treatment discontinuation was stronger in children treated with typical antipsychotics, such as haloperidol. This may reflect a narrower therapeutic window or higher side effect burden with these agents. Prior studies have shown that iron deficiency may exacerbate extrapyramidal side effects and diminish dopaminergic responsiveness, potentially reducing tolerability to typical antipsychotics (24).

It is worth noting that in our adjusted models, a single baseline ferritin measurement <30 ng/mL did not significantly predict discontinuation, highlighting the importance of temporal dynamics over static measurements. This supports the broader concept that variability in biological systems particularly at critical thresholds may have greater predictive power than absolute values (25). Such dynamic modeling has been increasingly applied in other domains, including metabolic (26) and cardiovascular research (27), where fluctuations in glucose or blood pressure predict outcomes more robustly than mean values.

Nonetheless, our findings must be interpreted within the context of several limitations. First, the retrospective nature of the study limits causal inference and may introduce unmeasured confounding. While we demonstrated the quantitative predictive value of serum ferritin, real-world treatment discontinuation is a complex multifactorial event. We lacked quantitative data on several other critical predictors, such as specific sociodemographic barriers (e.g., distance to healthcare facilities) and psychosocial tendencies (e.g., a tendency for caregivers to ignore mild symptoms that do not affect daily functioning). Furthermore, while we accounted for adverse events qualitatively as a reason for discontinuation, granular quantitative scales for adverse event severity were not uniformly available. Additionally, dietary iron intake, hepcidin levels, or markers of inflammation (e.g., interleukin-6) were not available. These factors can influence ferritin independently of iron status and may modulate the observed associations (28). Additionally, while ferritin is a widely used surrogate of iron stores, it is an acute-phase reactant and may be elevated in subclinical inflammation, although we attempted to mitigate this by excluding patients with CRP >5 mg/L. Furthermore, while our assessment suggested that differences in monitoring intensity did not materially confound our primary association, the requirement for serial measurements means our sample is derived from a clinically engaged population, which may affect generalizability. Future prospective studies should include a broader panel of iron-related markers, such as soluble transferrin receptor or hepcidin, to more accurately assess iron homeostasis.

A significant limitation of this study is the inherent selection bias stemming from its retrospective design. Since serial ferritin measurement is not standard clinical practice for all children with Tourette Syndrome, our study cohort inherently reflects a clinical selection process. The patients included were those for whom clinicians ordered serial tests based on specific diagnostic and management needs, for instance, to investigate symptoms such as motor restlessness suggestive of akathisia, fatigue, or nutritional inadequacy. These underlying clinical concerns could independently predispose patients to treatment discontinuation. This process introduces a bias whereby our sample may represent a subgroup with greater clinical complexity or a higher *a priori* risk for treatment non-adherence compared to the general TS population. Consequently, the observed association between ferritin fluctuations and antipsychotic discontinuation could be confounded by the underlying clinical factors that prompted the monitoring, and the generalizability of our findings should therefore be approached with caution.

Furthermore, the study was conducted at a single center, potentially limiting generalizability to other populations or healthcare settings. The sample size, though adequately powered for the primary analysis, limited subgroup explorations, especially for medication-specific interactions. Notably, adherence data were based on prescription refill records and clinical documentation, which may underestimate informal treatment discontinuation or caregiver-led dose modification.

Future research should aim to replicate these findings in a multicenter prospective cohort, ideally with integration of behavioral data, dietary assessments, and neuroimaging to explore mechanistic pathways. Given the dual association with both treatment persistence and symptom trajectory, intervention trials are strongly warranted to investigate whether stabilizing iron homeostasis through supplementation in children with high ferritin fluctuation can reduce discontinuation risk and improve clinical outcomes. Given the non-invasive and accessible nature of ferritin testing, this biomarker could become a routine component of treatment monitoring protocols in pediatric tic disorder management.

## Conclusion

This study provides novel evidence that dynamic fluctuations in serum ferritin, particularly below the critical threshold of 30 ng/mL, independently predict antipsychotic treatment discontinuation in children with tic disorders. This risk is further characterized by a stronger link to lack of efficacy and is associated with a less favorable symptom improvement trajectory. This highlights the potential of ferritin variability as a clinically actionable biomarker for treatment adherence risk stratification. Routine longitudinal ferritin monitoring may inform individualized care and timely iron interventions to optimize therapeutic engagement and outcomes.

## Data Availability

The raw data supporting the conclusions of this article will be made available by the authors, without undue reservation.
