# Supplementary material for: Serum ferritin critical threshold fluctuations predict antipsychotic treatment discontinuation in pediatric Tic disorders: a retrospective cohort study
Source: Front Pediatr. 2026 Jun 19;14:1826108. doi: 10.3389/fped.2026.1826108 (PMC13328395; doi:10.3389/fped.2026.1826108)
Supplement: Supplementary file 1 [file Table1.docx]

**Supplementary Table S1.** Baseline Characteristics Stratified by Mean Serum Ferritin Level

| **Characteristic** | **Low Mean Ferritin (<30 ng/mL)**  **(n=70)** | **Intermediate Mean Ferritin (30–50 ng/mL) (n=110)** | **High Mean Ferritin (>50 ng/mL)**  **(n=48)** | **Statistic** | **P-value** | **P for trend** |
| --- | --- | --- | --- | --- | --- | --- |
| Demographics |  |  |  |  |  |  |
| Age, years | 10.0 ± 2.8 | 10.4 ± 2.7 | 10.5 ± 2.5 | F=0.65 | 0.525 | 0.320 |
| Male sex | 52 (74.3%) | 78 (70.9%) | 35 (72.9%) | χ²=0.24 | 0.888 | 0.842 |
| Clinical Features |  |  |  |  |  |  |
| Tic subtype: |  |  |  | FET | 0.445 | - |
| - Tourette syndrome | 42 (60.0%) | 57 (51.8%) | 25 (52.1%) |  |  |  |
| - Chronic motor tic | 23 (32.9%) | 42 (38.2%) | 17 (35.4%) |  |  |  |
| - Chronic vocal tic | 5 (7.1%) | 11 (10.0%) | 6 (12.5%) |  |  |  |
| Baseline YGTSS total | 62.8 ± 11.8 | 55.9 ± 11.5 | 51.2 ± 10.9 | F=15.87 | <0.001 | <0.001 |
| Severe tics (YGTSS >60) | 45 (64.3%) | 40 (36.4%) | 10 (20.8%) | χ²=24.5 | <0.001 | <0.001 |
| Comorbidities: |  |  |  |  |  |  |
| - ADHD | 48 (68.6%) | 67 (60.9%) | 27 (56.3%) | χ²=2.28 | 0.320 | 0.143 |
| - OCD | 30 (42.9%) | 43 (39.1%) | 14 (29.2%) | χ²=2.45 | 0.294 | 0.120 |
| - Anxiety disorder | 17 (24.3%) | 21 (19.1%) | 8 (16.7%) | χ²=1.12 | 0.571 | 0.302 |
| - Depressive disorder | 8 (11.4%) | 10 (9.1%) | 4 (8.3%) | χ²=0.38 | 0.827 | 0.560 |
| Total psychiatric comorbidities (0/1/≥2) | 24/31/15 (34.3%/44.3%/21.4%) | 47/45/18 (42.7%/40.9%/16.4%) | 18/22/8 (37.5%/45.8%/16.7%) | χ²=2.89 | 0.823 | 0.716 |
| Positive family history | 26 (37.1%) | 35 (31.8%) | 13 (27.1%) | χ²=1.45 | 0.484 | 0.236 |
| Additional Covariates |  |  |  |  |  |  |
| Urban basic medical insurance | 44 (62.9%) | 78 (70.9%) | 36 (75.0%) | χ²=2.39 | 0.303 | 0.140 |
| Documented picky eating | 30 (42.9%) | 30 (27.3%) | 8 (16.7%) | χ²=9.89 | 0.007 | 0.001 |
| Outpatient visits, n | 9 [6-13] | 8 [5-12] | 8 [5-11] | H=3.78 | 0.151 | 0.075 |
| Iron Metabolism |  |  |  |  |  |  |
| Mean ferritin, ng/mL | 24.1 ± 3.8 | 39.2 ± 5.7 | 58.4 ± 6.9 | F=589.2 | <0.001 | <0.001 |
| Baseline ferritin, ng/mL | 26.3 ± 8.1 | 36.5 ± 12.0 | 47.8 ± 15.3 | F=45.21 | <0.001 | <0.001 |
| Hemoglobin, g/dL | 12.6 ± 1.2 | 13.2 ± 1.3 | 13.6 ± 1.5 | F=10.34 | <0.001 | <0.001 |
| MCV, fL | 82.9 ± 4.3 | 85.6 ± 4.5 | 87.8 ± 4.4 | F=18.24 | <0.001 | <0.001 |
| Treatment Parameters |  |  |  |  |  |  |
| Antipsychotic class: |  |  |  | χ²=0.85 | 0.653 | 0.498 |
| - Atypical | 58 (82.9%) | 94 (85.5%) | 41 (85.4%) |  |  |  |
| - Typical | 12 (17.1%) | 16 (14.5%) | 7 (14.6%) |  |  |  |
| Starting dose, mg/kg/day | 0.50 [0.33-0.73] | 0.48 [0.30-0.68] | 0.47 [0.30-0.65] | H=1.05 | 0.592 | 0.347 |

Values presented as mean ± SD, n (%), or median [IQR]. Abbreviations: YGTSS = Yale Global Tic Severity Scale; ADHD = attention-deficit/hyperactivity disorder; OCD = obsessive-compulsive disorder; MCV = mean corpuscular volume; FET = Fisher's exact test. Statistical tests: One-way ANOVA (F) or Kruskal-Wallis (H) for continuous variables; χ² or FET for categorical variables. Linear-by-linear association χ² test was used for trend (ordinal variables).

**Supplementary Table S2.** Baseline Characteristics Stratified by Intensity of Ferritin Monitoring

| **Characteristic** | **High-Intensity Monitoring (≥5 Measurements)**  **(n=112)** | **Low-Intensity Monitoring (≤4 Measurements)**  **(n=116)** | **Statistic** | **P-value** |
| --- | --- | --- | --- | --- |
| Demographics |  |  |  |  |
| Age, years | 10.5 ± 2.6 | 10.1 ± 2.8 | t=1.10 | 0.273 |
| Male sex | 83 (74.1%) | 82 (70.7%) | χ²=0.34 | 0.560 |
| Clinical Features |  |  |  |  |
| Tic subtype: |  |  | FET | 0.712 |
| - Tourette syndrome | 62 (55.4%) | 62 (53.4%) |  |  |
| - Chronic motor tic | 41 (36.6%) | 41 (35.3%) |  |  |
| - Chronic vocal tic | 9 (8.0%) | 13 (11.2%) |  |  |
| Baseline YGTSS total | 57.1 ± 12.3 | 55.7 ± 11.9 | t=0.89 | 0.375 |
| Severe tics (YGTSS >60) | 48 (42.9%) | 47 (40.5%) | χ²=0.13 | 0.717 |
| Comorbidities: |  |  |  |  |
| - ADHD | 73 (65.2%) | 69 (59.5%) | χ²=0.86 | 0.354 |
| - OCD | 42 (37.5%) | 45 (38.8%) | χ²=0.04 | 0.842 |
| - Anxiety disorder | 21 (18.8%) | 25 (21.6%) | χ²=0.29 | 0.591 |
| - Depressive disorder | 11 (9.8%) | 11 (9.5%) | χ²=0.01 | 0.931 |
| Total psychiatric comorbidities (0/1/≥2) | 45/48/19 (40.2%/42.9%/17.0%) | 44/50/22 (37.9%/43.1%/19.0%) | χ²=0.13 | 0.936 |
| Positive family history | 38 (33.9%) | 36 (31.0%) | χ²=0.22 | 0.639 |
| Additional Covariates |  |  |  |  |
| Urban basic medical insurance | 84 (75.0%) | 74 (63.8%) | χ²=3.91 | 0.048 |
| Documented picky eating | 32 (28.6%) | 36 (31.0%) | χ²=0.17 | 0.680 |
| Outpatient visits, n | 10 [7-14] | 8 [5-11] | U=4980 | 0.037 |
| Iron Metabolism |  |  |  |  |
| Mean ferritin, ng/mL | 35.2 ± 12.4 | 34.4 ± 11.5 | t=0.52 | 0.603 |
| Baseline ferritin, ng/mL | 36.0 ± 14.8 | 35.5 ± 13.7 | t=0.28 | 0.782 |
| Hemoglobin, g/dL | 13.2 ± 1.4 | 13.0 ± 1.4 | t=1.14 | 0.255 |
| MCV, fL | 85.5 ± 4.9 | 84.9 ± 4.7 | t=0.98 | 0.329 |
| Treatment Parameters |  |  |  |  |
| Antipsychotic class: |  |  | χ²=0.01 | 0.920 |
| - Atypical | 95 (84.8%) | 98 (84.5%) |  |  |
| - Typical | 17 (15.2%) | 18 (15.5%) |  |  |
| Starting dose, mg/kg/day | 0.49 [0.32-0.70] | 0.47 [0.30-0.68] | U=6165 | 0.401 |
| Study Exposure |  |  |  |  |
| High ferritin fluctuation | 40 (35.7%) | 38 (32.8%) | χ²=0.20 | 0.654 |

Values presented as mean ± SD, n (%), or median [IQR]. Abbreviations: YGTSS = Yale Global Tic Severity Scale; ADHD = attention-deficit/hyperactivity disorder; OCD = obsessive-compulsive disorder; MCV = mean corpuscular volume; FET = Fisher's exact test. Statistical tests: Independent t-test (normal variables), Mann-Whitney U test (non-normal variables), χ² or FET (categorical variables).

**Supplementary Table S3**. Estimated Marginal Means of YGTSS Scores at Key Timepoints by Ferritin Fluctuation Group

| **Timepoint** | **High-Fluctuation Group Mean**  **(95% CI)** | **Low-Fluctuation Group Mean**  **(95% CI)** | **Mean Difference**  **(High - Low)** | **95% CI for Difference** | **P-value for Difference*** |
| --- | --- | --- | --- | --- | --- |
| Baseline (Month 0) | 64.8 (61.9–67.7) | 52.3 (50.2–54.4) | 12.5 | (9.1, 15.9) | <0.001 |
| 6 Months | 55.2 (53.1–57.3) | 48.7 (47.0–50.4) | 6.5 | (3.8, 9.2) | <0.001 |
| 12 Months | 50.8 (48.3–53.3) | 42.4 (40.7–44.1) | 8.4 | (5.4, 11.4) | <0.001 |
| 18 Months | 46.3 (43.6–49.0) | 36.1 (34.0–38.2) | 10.2 | (6.8, 13.6) | <0.001 |
| 24 Months | 41.9 (38.7–45.1) | 29.8 (27.4–32.2) | 12.1 | (8.1, 16.1) | <0.001 |

*P-values for between-group differences at each timepoint were derived from the mixed model with Tukey adjustment for multiple comparisons. Note: Values are estimated marginal means (95% confidence intervals) derived from the linear mixed-effects model, adjusted for age, sex, antipsychotic class, and baseline YGTSS. The baseline values are model-estimated and closely match the observed means in Table 1.

**Supplementary Table S4.** Linear Mixed-Effects Model Parameters for Longitudinal YGTSS Scores

| **Model Term** | **β Coefficient** | **95% Confidence Interval** | **Standard Error** | **t-value** | **P-value** |
| --- | --- | --- | --- | --- | --- |
| Fixed Effects |  |  |  |  |  |
| Intercept | 58.34 | (54.21, 62.47) | 2.10 | 27.78 | <0.001 |
| Time (per month) | -0.92 | (-1.08, -0.76) | 0.08 | -11.50 | <0.001 |
| High Ferritin Fluctuation (vs. Low) | 4.18 | (1.12, 7.24) | 1.56 | 2.68 | 0.008 |
| Group × Time Interaction | 1.82 | (0.67, 2.97) | 0.59 | 3.09 | 0.002 |
| Age (per year) | -0.31 | (-0.72, 0.10) | 0.21 | -1.48 | 0.138 |
| Male sex (vs. Female) | 1.05 | (-0.98, 3.08) | 1.04 | 1.01 | 0.310 |
| Typical antipsychotic (vs. Atypical) | 2.11 | (-0.33, 4.55) | 1.24 | 1.70 | 0.090 |
| Baseline YGTSS (per 5 points) | 0.88 | (0.52, 1.24) | 0.18 | 4.89 | <0.001 |
| Random Effects | Variance | Standard Deviation |  |  |  |
| Participant Intercept | 45.2 | 6.72 |  |  |  |
| Residual | 32.7 | 5.72 |  |  |  |

Abbreviations: YGTSS, Yale Global Tic Severity Scale.
